# Effects of Various Caffeine Doses on Cognitive Abilities in Female Athletes with Low Caffeine Consumption

**DOI:** 10.3390/brainsci14030280

**Published:** 2024-03-15

**Authors:** Houda Bougrine, Achraf Ammar, Atef Salem, Khaled Trabelsi, Haitham Jahrami, Hamdi Chtourou, Nizar Souissi

**Affiliations:** 1 High Institute of Sport and Physical Education Gafsa, Gafsa University, Gafsa 2100, Tunisia; houdabougrine@live.fr; 2 Physical Activity, Sport and Health Research Unit (UR18JS01), National Observatory of Sports, Tunis 1003, Tunisiah_chtourou@yahoo.fr (H.C.); n_souissi@yahoo.fr (N.S.); 3 High Institute of Sport and Physical Education Sfax, University of Sfax, Sfax 3000, Tunisia; trabelsikhaled@gmail.com; 4 Department of Training and Movement Science, Institute of Sport Science, Johannes Gutenberg-University Mainz, 55099 Mainz, Germany; 5 Research Laboratory, Molecular Bases of Human Pathology, LR19ES13, Faculty of Medicine of Sfax, University of Sfax, Sfax 3029, Tunisia; 6 Interdisciplinary Laboratory in Neurosciences, Physiology and Psychology: Physical Activity, Health and Learning (LINP2), UFR STAPS (Faculty of Sport Sciences), Paris Nanterre University, 92000 Nanterre, France; 7 Research Laboratory, Education, Motricity, Sport and Health (EM2S), LR15JS01, High Institute of Sport and Physical Education of Sfax, University of Sfax, Sfax 3000, Tunisia; 8 Department of Psychiatry, College of Medicine and Medical Sciences, Arabian Gulf University, Manama 323, Bahrain; hjahrami@health.gov.bh; 9 Ministry of Health, Manama 410, Bahrain; 10 High Institute of Sport and Physical Education Ksar-Saïd, Manouba University, Mannouba 2010, Tunisia

**Keywords:** caffeine intake, dosages, low caffeine consumption, female athletes, side effects, team ball athletes, cognitive performance

## Abstract

Caffeine (CAF), a prevalent psychoactive stimulant, is believed to potentially enhance cognitive ability. However, studies on the effects of various doses are limited and yield inconsistent results, particularly in female athletes. Therefore, we aimed to assess the association between three different dosages of CAF intake (low, moderate, and high) and cognitive skills in female athletes with low CAF consumption. This study had a randomized, crossover, double-blind design in which each athlete performed four experimental sessions after ingesting either a placebo (PLAC), 3 mg·kg^−1^ of CAF (3 mg of CAF), 6 mg·kg^−1^ of CAF (6 mg of CAF), or 9 mg·kg^−1^ of CAF (9 mg of CAF) with an in-between washout period of at least 72 h. Following a 60 min window post-capsule consumption, fourteen female athletes (age: 17.4 ± 0.8 years) were assessed through various cognitive tests, namely, simple reaction time (SRT), choice reaction time (CRT), and attentional task (AT) tests, along with the mental rotation test (MRT). Additionally, they were required to complete a questionnaire about the undesirable side effects of CAF. Our results indicated that, compared to those of PLAC, the SRT, CRT, and AT performance were significantly improved following the administration of both 3 mg of CAF and 6 mg of CAF. While the greatest enhancement was observed after consuming _3_ mg of _CAF_, no significant differences were found between the effects of 3 mg and 6 mg of CAF. Interestingly, MRT performance did not improve with any of the CAF dosages. Moreover, the ingestion of 9 mg of CAF did not enhance cognitive skills and was linked to the highest occurrence of CAF-related side effects. In conclusion, our results highlight the recommendation for a low CAF dosage of 3 mg·kg^−1^, in contrast to a higher dose of 6 mg·kg^−1^ or 9 mg·kg^−1^ of CAF, to enhance various aspects of cognitive performance in female athletes with low CAF consumption without adverse side effects.

## 1. Introduction

Over the last decade, mental health awareness has surged, evolving to be recognized not merely as the absence of illness but as essential for managing life’s daily demands and stress [1]. Moreover, the role of nutrition in enhancing physical and cognitive functions has been underscored as key to our development [2]. The link between diet and better physical and mental performance is assumed to be of paramount importance and is especially crucial for elite athletes. For instance, in Olympic competitions, where the smallest margins can determine victory, a mere 1–2% boost in performance may be the decisive factor in winning gold [3]. For athletes operating at their optimal genetic potential, this necessitates a strategically planned approach to training, recovery, and nutrition, exploiting the full array of scientific knowledge to gain that critical edge [4]. Optimal nutrition affects not only physical attributes but also psychological parameters and cognitive processing, which are essential for late-game performance in team sports where fatigue can impair focus and decision-making abilities [5].

Cognitive abilities comprise a diversity of mental processes, including aspects such as executive function and decision-making [6,7]. The unequivocal significance of these cognitive abilities in sports has recently led to increased interest in deciphering the complex relationship between ergogenic aids such as caffeine (CAF) consumption and its effects on cognitive performance during athletic exercise. Recent findings suggest that consuming a low to moderate amount of CAF immediately before or during physical exercise can enhance cognitive processes, including attention, while simultaneously boosting energy levels and improving mood [8]. Additionally, this CAF intake can enhance simple and choice reaction times, boost memory, and counteract fatigue [8]. CAF, a widely consumed beverage worldwide, is second only to water and has emerged as the most intensely scrutinized supplement in sports research, with studies investigating its influence on athletic performance witnessing a significant surge in recent years [9]. CAF (1,3,7-trimethylxanthine) is widely recognized as a supplement frequently utilized by athletes across a diverse range of sports disciplines [10,11]. The widespread use of CAF supplements among athletes, commonly taken before or during competitions [12,13], can be attributed to the findings of numerous studies confirming the potential positive impacts of CAF supplements on sports performance [14,15]. A key mechanism of CAF ergogenicity is its antagonistic impact on adenosine receptors due to its similar chemical structure to adenosine [16,17]. This antagonistic action enables an increase in dopamine release, inducing alertness [18,19], while also boosting the concentration of other central nervous system neurotransmitters that adenosine typically suppresses [17,18]. However, studies exploring the impacts of CAF supplementation on cognitive function in athletes are limited in scope and have produced conflicting results [8]. In female team sports players, CAF (6 mg·kg^−1^) was found to enhance vigor and reaction time, but it did not significantly impact the results of the Stroop test [20,21]. Furthermore, evidence suggests that a dosage of 5 mg·kg^−1^ of CAF can improve cognitive performance after an arm-based Wingate test [22]. However, interestingly, the acute intake of an identical dosage (5 mg·kg^−1^) of CAF might detrimentally affect the performance of professional soccer players, particularly in the computerized Stroop test [23]. A dose of 3 mg·kg^−1^ of CAF did not affect the multiobject tracking ability of active men [24]. Moreover, CAF supplements at doses of 2 and 4 mg·kg^−1^ did not impact reaction or target tracking times during clay target shooting [25], suggesting that CAF intake likely does not affect tracking capabilities in sports. Thus, a recent study highlighted that a dose of 3 mg·kg^−1^ of CAF intake did not affect soccer players’ short-pass accuracy but varied their long-pass performance and decreased their decision-making scores [26]. These inconsistencies may be due to the diverse athletic populations examined and differences in both the method and dosage of CAF administration. Ultimately, the variability in performance enhancement resulting from different CAF dosages among diverse studies and participants complicates the comparison of the ergogenic effects of low and high CAF doses. Consequently, additional research is necessary to examine the impact of various CAF doses on the same individuals, enabling more accurate comparisons and stronger conclusions. However, the potential negative side effects of CAF consumption, particularly at the doses taken to enhance performance, have received relatively little attention [8,27].

Research gaps persist in understanding the relationship between CAF dosage and performance, especially in female athletes. There is an apparent scarcity of focused research in this area, as evidenced by female participants only making up 13% of participants in 362 exercise-related CAF studies [15]. Furthermore, a recent meta-analysis examining the effects of CAF on cognitive functions included only a limited number of studies concerning female athletes [8]. This highlights an important research oversight, suggesting a significant gap in understanding how CAF dosage specifically affects this demographic. Furthermore, the results from male groups may not apply to females, requiring the need for sex-differentiated studies [28]. Thus, detailed guidelines for female athletes, accounting for potential sex differences, are crucial.

The purpose of this study was to examine the effect of various dosages of CAF supplementation on different cognitive skills and the prevalence and severity of side effects associated with CAF supplementation in female athletes with low CAF consumption. We hypothesize that the ingestion of CAF at a low dosage could mirror the effects observed at a moderate dosage, thereby enhancing executive functions.

## 2. Materials and Methods

### 2.1. Participants

In accordance with the guidelines proposed by Beck [29], we utilized G*Power software (version 3.1.9.6; Kiel University, Kiel, Germany) [30] to pre-determine our sample size. We set a significance level (α) of 0.05 and aimed for a statistical power (β) of 0.95. Drawing on the findings of Karayigit et al. [31], which indicated a medium effect of CAF ingestion on reaction time among female athletes (ηp^2^ = 0.15, resulting in an effect size f = 0.42), and after discussions among the authors, we estimated the effect size to be f = 0.42. Consequently, a minimum of 14 athletes was required to achieve the desired statistical power and minimize the risk of a type II error. Out of the 41 surveys reviewed, 26 female team ball sports players were deemed eligible to take part in the study. However, three participants dropped out, and nine participants were excluded due to their menstrual cycle, while fourteen participants completed the entire study protocol and were subsequently included in our analyses. All of these female participants (mean (SD) age 17.4 ± 0.8 y; height 1.63 ± 0.1 m; body mass 59.7 ± 9 kg; BMI 22.3 ± 1.9 kg·m^2^) met the following criteria: (a) aged 15 to 20 years; (b) had a daily CAF intake of less than 0.99 mg·kg·day^−1^ (low consumers) but higher than 25 mg/day^−1^ (not naïve consumers) [32]; (c) had active participation in team ball sports for at least 3 years, a minimum of 3 times/week for the last 6 months; (d) had not consumed any medications, dietary supplements, or performance-enhancing aids that could impact the study results; (d) had regular menstrual cycles with a swing of no more than 3 days over the last 4 months [33]; and (f) had a regular sleep duration. Participants were excluded from the study if they (a) had extreme chronotypes; (b) had positive alcohol and/or smoking status; (c) had a potential allergy to CAF; (d) had a daily CAF intake of less than 25 mg·day^−1^ (classified as naive CAF consumers); (d) had a history of a menstrual disorder within the past four months and/or had taken oral contraceptives or medications in the previous four months, including pills, patches, injections, implants, and intrauterine devices; and (d) had a history of disease and/or the use of any medications for any chronic medical condition. All the athletes came to the study with a minimum of three years’ experience (5.4 ± 0.9 years) of team ball sports, and they had consistently engaged in at least three training sessions weekly (4.4 ± 0.5 sessions/week) over the past six months. The participants were categorized as low consumers of CAF (42.4 ± 9.4 mg·day^−1^ or 0.7 ± 0.2 mg·kg·day^−1^), with daily consumption ranging between 25 mg·day^−1^ and 0.99 mg·kg·day^−1^, following a previously suggested classification [32]. The chronotypes of the athletes were determined using the Horne and Ostberg [34] questionnaire, which assesses sleep and activity preferences to mitigate circadian influences on the study. Only participants with intermediate chronotypes (49.5 ± 4.5) were included in our analysis. Sleep quality was assessed during the month preceding the experiment by the PSQI, which averaged approximately 7.8 ± 0.6 h of sleep. Menstrual cycle length (27.7 ± 1.9 days) and phases were tracked using the My calendar^®^ Period Tracker, focusing on evaluations during the follicular and luteal phases [35]. Athletes and their parents received all explanations about the experiment’s timeline, the tasks involved, and assessment processes before they agreed to participate in writing. Before participating in the study, all participants and their parents provided written consent. All protocols and methods received approval from the Local Research Ethics Committee of the University of Jendouba (054-2023), adhering to the recent edition of the Declaration of Helsinki.

### 2.2. Experimental Design

**Figure 1:**
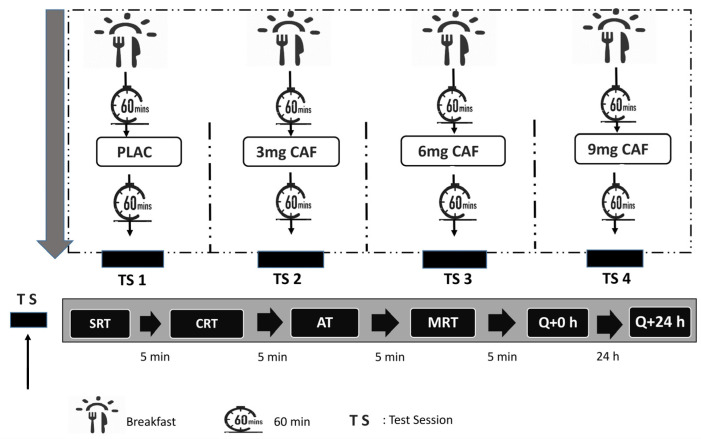
Study design. TS: test session; PLAC: cellulose; 3 mg CAF: 3 mg·kg^−1^ of CAF; 6 mg CAF: 6 mg·kg^−1^ of CAF; 9 mg CAF: 9 mg·kg^−1^ of CAF; SRT: simple reaction time; CRT: choice reaction time; MRT: mental rotation test; Q: side effects of CAF intake questionnaire.

The study was conducted using a randomized, double-blind, crossover design. Participants were randomized in each condition using an online software tool (http://www.jerrydallal.com/random/randomize.htm (accessed on 20 January 2023)). Before the experiment, participants were familiarized with the process, which involved assessing anthropometric indices, cognitive tests, and questionnaires to reduce learning effects during the experiment and guarantee high-quality results. This was followed, as illustrated in Figure 1, by four identical experimental sessions spaced at least 72 h apart to ensure adequate washout. These sessions varied only in the substance consumed: a PLAC, 3 mg·kg^−1^ of CAF (3 mg of CAF), 6 mg·kg^−1^ of CAF (6 mg of CAF), or 9 mg·kg^−1^ of CAF (9 mg of CAF).

Sixty minutes before beginning the testing procedures, participants ingested opaque capsules. These capsules contained specific substances intended to reach peak plasma CAF concentrations within the timeframe. To ensure the integrity of the experiment, blinding and randomization tasks were performed by a research team member who was not engaged in the direct collection of the data. Subsequently, participants completed a battery of cognitive tests in a predefined sequence. The tests included the simple reaction time test (SRT), choice reaction time test (CRT), attention task test (AT), and mental rotation test (MRT) in each experimental session. To facilitate adequate recovery, a five-minute rest interval was allowed between each test. Participants were permitted to depart upon the appropriate completion of all tests.

Participants were asked to refrain from CAF intake and strenuous exercise 24 h before each trial. They were provided with a list of food and drink items containing CAF and asked to avoid consuming these items for the entire testing day and the 24 h preceding each test session. To replicate these conditions, participants were asked to record their activities 24 h before the first trial and follow the same pattern before the second, third, and fourth trials. The records were subsequently photocopied, with each athlete receiving copies to ensure dietary consistency across future sessions. Before commencing each session, participants verbally confirmed adherence to the established dietary guidelines and confirmed the avoidance of any excluded substances containing CAF. Participants affirmed that they maintained their usual lifestyle habits, training, sleep, hydration, and dietary routines throughout the study period and ensured consistency in preparation for each study visit, particularly in the 24 h leading up to each visit. Body mass was consistently recorded during the morning sessions throughout the various familiarization sessions, utilizing an electronic scale provided by Tanita (Tokyo, Japan) to calculate the appropriate CAF dosage for each condition. Participants were instructed to defecate and urinate before the weighing process to ensure the accuracy of the body mass assessment [36].

To maintain consistency across the study, each testing session was carried out under identical conditions. The same indoor court was used, with all equipment and testing sequences being uniform and under the continual supervision of the same research team. The ambient temperature and relative humidity were approximately 28 °C and 46%, respectively, ensuring that the environmental factors remained consistent throughout the four experimental sessions. Furthermore, to mitigate the impact of circadian rhythms on the outcomes and ensure consistency in the conditions under which the tests were performed, the experiment was meticulously scheduled, with all four trials consistently commencing at 09:00 AM [37,38].

Participants were administered capsules containing either a dose of CAF (CAF) (Bulk Powders, Colchester, UK) or a placebo consisting of an inert substance (Cellulose; Guinama 6, Valencia, Spain). These were ingested in a double-blind design to preserve the study’s integrity. To facilitate absorption, the capsules were taken with 10 µL of water one hour before the trial commencement and at least one hour after the last meal, ensuring consistency in the absorption period. These measures are crucial for aligning with the known pharmacokinetics of CAF; specifically, peak plasma concentrations are typically achieved between 15 and 120 min after oral intake, as outlined by Magkos and Kavouras [39]. The precise timing of 60 min before testing aligns with findings from Chia et al. [40], which indicate that CAF achieves maximum plasma levels roughly an hour following consumption, facilitated by swift gastrointestinal absorption. To mitigate any potential gastrointestinal discomfort and align with the fasting requirements, participants were provided with a standardized breakfast of approximately 500 kcal, which was consumed 120 min before each trial, departing from their usual pretrial nutritional regimen [21].

#### 2.2.1. Habitual CAF Intake Assessment

The evaluation of the participants’ regular CAF consumption was accomplished using a modified version of the Food Frequency Questionnaire (FFQ) suggested by Bühler et al. [41]. The quantity of food ingested daily was determined individually using household measurements for the month preceding the commencement of the study, in line with earlier recommendations [32]. An experienced nutritionist utilized nutritional tables for database construction to calculate the daily CAF intake for each athlete during the four weeks leading up to the experimental trials, considering the body mass of each athlete and not only the daily intake of CAF, as suggested by Filip et al. [32]. To ensure homogeneity in our sample and to avoid discrepancies in classification that could affect our data analysis, only low CAF consumer athletes with daily CAF intake ranging between 25 mg·day^−1^ and 0.99 mg·kg·day^−1^ were included in this study.

#### 2.2.2. Simple Reaction Time (SRT)

The processing speed was evaluated through the simple reaction time (SRT) method. Instructions were given to the participants to quickly hit a button when they perceived a visual signal on the computer screen. SRT was conducted using the Reaction, INRP-free software (version 4.05) developed by Tilquin.

#### 2.2.3. Choice Reaction Time (CRT)

Using the same software, participants were presented with a colored geometric shape, termed the “target”. Following this, they were shown a sequence of geometric forms of varying hues. The participants were directed to hit a button as swiftly as possible each time they identified the target form. The software was designed to evaluate the reaction time from the moment the form was displayed to the participant’s response. A higher score represented a slower response, indicating a less optimal performance.

#### 2.2.4. Attention Task (AT)

The attention task (AT), also known as the number cancellation test, is a tool used to evaluate cognitive–perceptual motor functioning, psychomotor speed, and the ability to maintain prolonged attention [42]. The NRS-2002 aims to provide a practical and reliable evaluation of various aspects of prefrontal cortex functions, which include but are not limited to sustaining attention, information processing speed, and executive functioning [43]. The AT comprises four pages, each filled with 600 numbers ranging from one to five digits, neatly organized into 36 lines. During the test, participants were given 1 min to cross out as many three-digit numbers as possible. A total of 187 targets were evenly distributed across the pages, with 2 to 8 three-digit numbers randomly placed in each line. Each number was set apart by a dot and had a space before and after it. The participants’ score, derived from awarding 1 point for each correctly identified target (max 187), served as an indicator of their attention span [43].

#### 2.2.5. Mental Rotation Test (MRT)

Tasks related to mental rotation present participants with an image featuring two distinct objects. The challenge lies in discerning whether these objects match. Although one object may be deliberately positioned differently than its counterpart, individuals are required to employ their cognitive abilities to visualize and “mentally rotate” the object in question to confirm its resemblance to the other [44]. The MRT was carried out utilizing Open Sesame software, version 3.1 [45]. Every test set was composed of 10 elements reflecting participants’ demanding mental focus, accuracy, and quick cognitive flexibility. When examining the results of the MRT, two distinct types of scores emerge as key indicators of performance. The first of these is the MRT time, which refers to the duration taken by the participant to correctly identify and process the items presented. This serves as a measure of the athlete’s mental agility and speed of cognitive processing. The second score, MRT errors, is a record of the number of errors made by the participant throughout the test.

#### 2.2.6. Undesirable Side Effects of the CAF Intake Questionnaire

Following the completion of the cognitive tests, participants were asked to fill out a questionnaire focused on the negative side effects linked to CAF intake (e.g., urine output, gastrointestinal problems, tachycardia, or headache) immediately. The same questionnaire was also administered once again the following morning. This survey contained eight items with a yes/no scale, as established by Pallarés et al. [46], enabling a systematic evaluation of the side effects of CAF intake.

### 2.3. Statistical Analysis

All the statistical analyses were performed using STATISTICA 10 software (StatSoft, Paris, France). The calculation of the mean and standard deviation (SD) was carried out for each variable. The Shapiro–Wilk test confirmed the normal distribution of all the data sets. The impact of the CAF supplementation dosage was analyzed using one-way repeated-measures ANOVA (4 Testing doses). In instances where significant differences existed between means, Tukey’s HSD post hoc test was used for verification. Effect sizes were assessed using the effect size statistic (ηp2), with 0.01 indicating a small effect size, 0.06 representing a moderate effect size, and 0.14 illustrating a large effect size, as per the criteria outlined by Cohen [47]. Standardized effect size (Cohen’s d) analysis was used to determine the magnitude of differences between variables according to previous methods [48]: trivial (d ≤ 0.20); small (0.20 < d ≤ 0.60); moderate (0.60 < d ≤ 1.20); large (1.20 < d ≤ 2.0); very large (2.0 < d ≤ 4.0); and extremely large (d > 4.0). A significance level was set at *p* ≤ 0.05.

## 3. Results

### 3.1. Simple Reaction Time (SRT)

**Figure 2:**
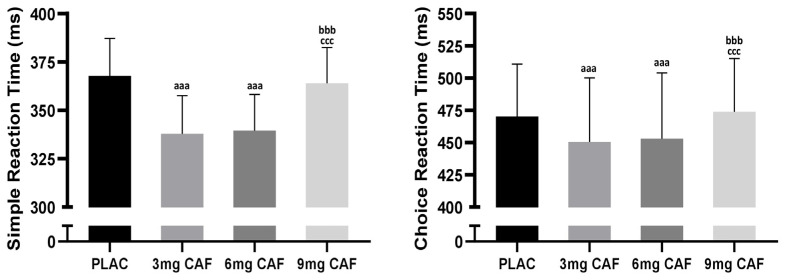
Mean ± SD values of simple reaction test (SRT) and choice reaction test (CRT) results reported after the administration of cellulose (PLAC), 3 mg·kg^−1^ of CAF (3 mg of CAF), 6 mg·kg^−1^ of CAF (6 mg of CAF), or 9 mg·kg^−1^ of CAF (9 mg of CAF). ^aaa^ (*p* < 0.001): significant difference compared to the placebo group. ^bbb^ (*p* < 0.001): significant difference compared to 3 mg of CAF. ^ccc^ (*p* < 0.001): significant difference compared to 6 mg of CAF.

ANOVA revealed that there were significant main effects of CAF dose on SRT (F (3, 39) = 40.46; *p* < 0.001; np2 = 0.75). The post hoc test demonstrated that the dosages of 3 mg of CAF (−8.2%, *p* < 0.001) and 6 mg of CAF (−7.7%, *p* < 0.001) improved SRT compared to that of PLC, but there was no significant difference between the two dosages (*p* > 0.05). The ingestion of _9_ mg of _CAF_ was not significantly different between SRT and PLC (*p* > 0.05) (Figure 2).

### 3.2. Choice Reaction Time (CRT)

A significant main effect of CAF dose (F (3, 39) = 27.69; *p* < 0.001; np2 = 0.68) on CRT was observed. While CRT performance remained unaffected after 9 mg CAF (*p* > 0.05) intake, the ingestion of both 3 mg of CAF (−4.2%, *p* < 0.001) and 6 mg of CAF (−3.7%, *p* < 0.001) enhanced CRT compared to PLC. Furthermore, no significant difference in CRT efficacy was detected between 3 mg of CAF and 6 mg of CAF (*p* > 0.05, Figure 2).

### 3.3. Attention Task (AT)

**Figure 3:**
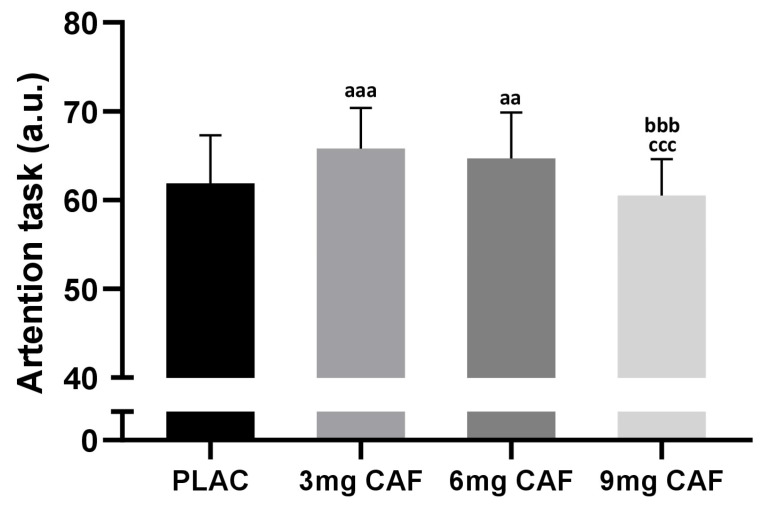
Mean ± SD values of attention task (AT) scores reported after the administration of cellulose (PLAC), 3 mg·kg^−1^ of CAF (3 mg of CAF), 6 mg·kg^−1^ of CAF (6 mg of CAF), or 9 mg·kg^−1^ of CAF (9 mg of CAF). ^aaa^ (*p* < 0.001), ^aa^ (*p* < 0.01): significant difference compared to the placebo group. ^bbb^ (*p* < 0.001): significant difference compared to 3 mg of CAF. ^ccc^ (*p* < 0.001): significant difference compared to 6 mg of CAF.

We found a significant effect for AT (F (3, 39) = 23.74; *p* < 0.001; np2 = 0.64). AT scores increased significantly after the administration of 3 mg of CAF (6.4%, *p* < 0.001) or 6 mg of CAF (4.6%, *p* < 0.01) in comparison with those in the PLAC condition but not after the intake of 9 mg of CAF (*p* > 0.05). Moreover, no significant differences in AT performance were revealed between 3 mg CAF and 6 mg CAF (*p* > 0.05) dosages (Figure 3).

### 3.4. Mental Rotation Test (MRT)

#### 3.4.1. MRT Time

**Figure 4:**
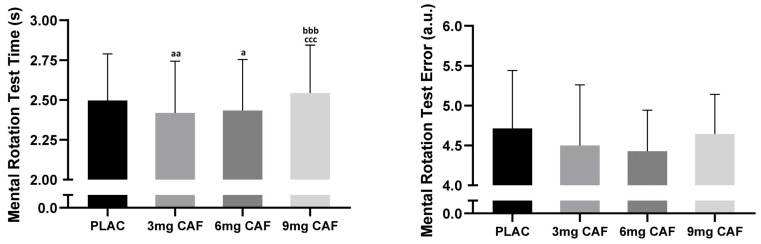
The mean ± SD values of the mental rotation test (MRT) data, including MRT time and MRT errors, were reported after the administration of cellulose (PLAC), 3 mg·kg^−1^ of CAF (3 of mg CAF), 6 mg·kg^−1^ of CAF (6 mg of CAF), or 9 mg·kg^−1^ of CAF (9 mg of CAF). ^aa^ (*p* < 0.01), ^a^ (*p* < 0.05): significant difference compared to the placebo. ^bbb^ (*p* < 0.001): significant difference compared to 3 mg of CAF. ^ccc^ (*p* < 0.001): significant difference compared to 6 mg of CAF.

One-way ANOVA demonstrated significant effects (F (3, 39) = 13.36; *p* < 0.001; np2 = 0.50) of CAF dosage on MRT time, indicating that CAF dosage interferes with MRT time. Compared to that of the PLAC condition, the MRT time was better after the ingestion of 3 mg of CAF (−3.1%, *p* < 0.01) or 6 mg of CAF (−2.5%, *p* < 0.05). However, no difference was detected after the ingestion of a higher dose of _9_ mg of _CAF_ compared to that of the PLAC either between low or moderate doses (3 mg of CAF vs. 3 mg of CAF) (both *p* > 0.05 (Figure 4).

#### 3.4.2. MRT Errors

One-way ANOVA revealed that there were no significant effects on MRT errors (F (3, 39) = 1.00; *p* = 0.40; np2 = 0.07) regardless of the dose of CAF (Figure 4).

### 3.5. Undesirable Side Effects of the CAF Intake Questionnaire

Immediately following the PLAC trials, the incidence of side effects was low, ranging from nonexistent to 7.14% posttrial (Q + 0 h). Regarding varying CAF dosages, minor side effects such as muscle and gastrointestinal discomfort were infrequently reported in the 3 mg of CAF (0–14.28%) and 6 mg of CAF (0–21.42%) trials during Q + 0 h. On the other hand, the 9 mg CAF dose led to a greater occurrence of undesirable symptoms, including tachycardia and heart palpitations (35.71%), headache (21.42%), and gastrointestinal problems (28.57%), affecting approximately 0% to 35.71% of the participants (Q +0 h).

The day following each test (Q + 24 h), side effects were generally minimal across the PLAC (0–7.14%; Q + 0 h), 3 mg of CAF (0–14.28%; Q + 0 h), and 6 mg of CAF (0–14.28%; Q + 0 h) conditions. Notably, 24 h after _9_ mg of _CAF_ intake, there were more reports (7.14–35.71%) of increased urine output (35.71%), tachycardia and heart palpitations (35.71%), headache (28.57%), and gastrointestinal problems (35.71%) (Table 1). Interestingly, when participants were asked about the order of supplementation following the trials, they correctly identified their intake as 3 mg of CAF in only 14.28% (2 out of 14) of the related trials. The accuracy was slightly greater for the 6 mg of CAF and 9 mg of CAF trials, with correct identification occurring in 21.42% of the instances for both dosages. Furthermore, even in placebo trials, 14.28% of the participants were able to accurately recognize that they had taken a placebo (2 out of 14). Despite these findings, no athlete (0 out of 14) successfully pinpointed the exact four trials they participated in, indicating that the blinding within the study was maintained.

## 4. Discussion

To the best of our knowledge, this is the first study in which the effects of three different doses of CAF, in the form of a capsule, on SRT, CRT, attention, MRT, and CAF adverse effects on female athletes were investigated. The main finding of the current study was that, compared with placebo, both lower (3 mg of CAF) and moderate (6 mg of CAF) doses of CAF improved reaction times and attention tasks, with more trivial enhancements observed under the lower dose. However, regardless of the dosage, the CAF did not significantly affect the MRT. A higher CAF dose did not enhance cognitive performance but was associated with a greater incidence of adverse effects.

Reaction times and attention performance were enhanced after CAF ingestion in our study, which aligns with the findings of a recent meta-analysis indicating that the acute consumption of CAF in low to moderate amounts before or during physical activity can enhance cognitive performance, such as alertness, response time, and decision-making [8]. In addition, our findings are consistent with prior studies suggesting that, regarding cognitive performance, smaller doses have demonstrated superior outcomes [19,49,50]. This is evidenced by Kaplan et al. [49], who observed that, compared with larger doses, smaller doses of CAF produced more favorable outcomes in a reaction time test. This finding implies that a low CAF dosage might be associated directly and specifically with increased speed or efficiency in perceptual motor tasks [19]. Furthermore, a previous study reported that even relatively low doses of CAF (i.e., 12.5, 50, or 100 mg) improved simple reaction time performance compared to a placebo [51]. Biggs et al. [52] reported similar beneficial effects in a nonathletic population, where a dose as low as 100 mg enhanced performance in a driving stimulation test. To explain this, it has been recently proposed that lower doses, not higher doses, act as antagonists of adenosine receptors, while higher doses employ different modes of action to induce ergogenic effects [53].

However, regarding moderate doses, the results are inconclusive. Duncan et al. [22] reported that a moderate CAF dose (5 mg·kg^−1^) could enhance cognitive performance after a Wingate test with arms. However, these results contrast with those of other studies in which the same dosage of CAF did not significantly impact Stroop test performance in rested participants [54,55]. De Almeida et al. [23] reported that a soccer-specific exercise protocol improved Stroop test performance in professional soccer players, but acute CAF ingestion (5 mg·kg^−1^) was detrimental. Moreover, Bougrine et al. [21] and Ali et al. [20] reported an increase in reaction time and selective attention after the administration of 6 mg·kg^−1^ of CAF to female athletes.

By comparing various dosages of CAF within the same population, Karayigit et al. [31] revealed the advantageous effects of two different doses of CAF (3 and 6 mg·kg^−1^) on female athletes on the flanker task. However, Zhang et al. [56] found contrasting evidence. Their study showed that a lower CAF dose of 3 mg·kg^−1^ boosted Stroop task performance in male athletes, while higher doses (6 and 9 mg·kg^−1^) did not yield similar benefits. This finding suggested the potential dose dependency of the effectiveness of CAF and the potential influence of sex.

The variability in results across different studies may be due to several key factors. These included the specific dosage of CAF administered, the supplementation protocol (the washout period and trial timing), the sex of the participants, the form in which the CAF was supplemented, and the state of the subjects at the time of the trials, specifically, whether they were rested or fatigued. These diverse findings could provide a comprehensive explanation for the observed discrepancies in outcomes. Furthermore, these discrepancies among studies could be attributed to individual daily CAF consumption habits and their interaction with the administered dosage. Recent classifications of CAF consumers present a unique perspective, suggesting that individuals consuming low daily amounts of CAF (25–99 mg·kg·day^−1^) do not fall into the categories of naive (<25 mg·day^−1^) or mild consumers (1.00–2.99 mg·kg^−1^) [32]. This distinction holds considerable weight in data interpretation, as studies often categorize their subjects only as low or moderate CAF consumers. However, a ‘low consumer’ could be further classified as CAF-naive, low, or mild in light of recent, rigorous categorization criteria. Moreover, the data on the effects of low-to-moderate CAF intake in healthy individuals, including sex-specific impacts, remain notably inconclusive and limited. Considering the potential mechanisms underlying the effectiveness of CAF, its molecular structure, similar to that of adenosine, allows it to attach themselves to adenosine receptors found within brain tissue. As a result, it mitigates the adverse impacts of the fat-related intensification of the nervous system caused by adenosine [57]. For example, CAF inhibits the A2a receptor in the striatum, which in turn stimulates the D2 dopamine receptor and amplifies the triggering influence of dopamine on psychomotor activity [57].

Regarding the impact of different dosages of CAF, we hypothesize that the effects of CAF are dose-dependent, a finding that aligns with those of numerous previous studies [19,58,59,60]. The effect of CAF dosage has been likened to an inverted “U”, with both the smallest and largest doses leading to mediocre performances [19,50]. At lower consumption levels (i.e., >300 mg/day, equivalent to two to three cups of coffee), CAF is unlikely to have adverse effects [50] and may even enhance performance [59]. McLellan et al. [59] proposed that CAF plasma concentrations in the range of 15 to 20 μM could enhance performance. However, lower concentrations may not yield beneficial effects on performance. Intriguingly, it has been shown that the peak plasma CAF concentration does not necessarily coincide with the peak performance [61]. Conversely, despite its low plasma concentration, a moderate dose of CAF (5 mg·kg^−1^) retains its ergogenic effects several hours after consumption [62]. The widely accepted theory about the effects of CAF centers around its proximity to adenosine and its ability to act as an antagonist of adenosine receptors [63]. This interaction is believed to be the reason that CAF can bind to these receptors, leading to behaviors indicative of alertness, including heightened attention, improved mood, and increased arousal in an animal model [63]. Furthermore, our findings showed that a higher CAF dosage did not result in improved cognitive function. We suggest that this lack of cognitive enhancement at higher dosages, which is in line with previous study results [56], may be attributed to the onset of CAF-induced side effects, which include gastrointestinal discomfort, confusion, diminished concentration ability, and nervousness [64]. Research into the effects of CAF on cognitive performance in female athletes has yielded notable findings. Ali et al. [20] observed that a dose of 6 mg·kg^−1^ of CAF tended to reduce perceived exertion and improve reaction times in both the Stroop and CRT tests. Similarly, the consumption of caffeinated coffee, with doses of 3 and 6 mg·kg^−1^ of CAF, was associated with improved reaction time and response accuracy in cognitive tasks among 17 female athletes after consuming doses of 3 and 6 mg·kg^−1^ of CAF [31]. Stojanović et al. [65] reported that an intake of 3 mg·kg^−1^ of CAF among female basketball players led to a moderate, yet significant, decrease in RPE during a physical test battery. Furthermore, Bougrine et al. [21] demonstrated that the ingestion of 6 mg·kg^−1^ of CAF improved reaction times and attention task performance among female handball players. Considering that a mere 13% of the studies currently available in the literature focus on women, it becomes clear that any recommendations concerning CAF usage cannot be applied directly or accurately to female athletes [15]. This highlights the necessity for further investigation into the effects of CAF on cognitive performance, particularly in female athletes. Indeed, there is a clear need for additional studies in this area, given that the current research landscape is notably focused on exploring the physical impact of CAF rather than its cognitive effects.

On the other hand, our findings demonstrated that, regardless of dosage, CAF did not significantly affect or enhance performance on the mental rotation test, which is a more complex task than other tests. We hypothesize that the complexity of the mental rotation task, which requires more focus and cognitive processing, may explain why CAF did not enhance performance on this test, unlike simpler tasks such as attention and reaction time tasks. Indeed, there is a positive consensus regarding the impact of CAF on simpler aspects of cognition, including simple and choice reaction times and attention. CAF intake has been found to enhance feelings of alertness and activeness [6], as well as reaction speed [66] and accuracy [67]. However, the effect of CAF on more complex cognitive tasks appears to be contentious. Numerous studies suggest that CAF can improve focus [68], problem-solving abilities requiring logical reasoning [69], cognitive function, and neuromuscular coordination [52,70,71,72]. Nonetheless, the administration of CAF did not yield any effects during a marksmanship exercise using an unarmed rifle, a complex task requiring target detection, fine motor coordination, or stability [72]. Moreover, studies have investigated the effects of different quantities of CAF on cognitive abilities, particularly reaction target tracking times in clay target shooting and object tracking in sports [24,25]. Furthermore, no significant impact on Stroop test performance was revealed after CAF ingestion [54,55]. A recent review suggested that CAF offers only a limited ability to enhance mental performance in a sports setting [73]. Although its effects are consistent, they are restricted, and CAF provides little benefit in terms of enhancing spatial or verbal working memory or executive function [73] which is in line with our results. While the psychological effects of CAF are consistently linked to increased subjective alertness and improved performance in tasks assessing attention or focused attention, its benefits are limited to these areas [8,51,74,75]. CAF’s effects generally do not extend to other cognitive domains relevant to sports [73]. CAF consumption has not demonstrated any significant impact on long-term memory tasks and shows inconsistent effects on working memory and executive function tasks, potentially impairing the performance of more complex tasks [19,76]. However, recent data suggest that CAF’s cognitive-enhancing effects may expand into more complex cognitive tasks under conditions of significant sleep loss or restriction [77].

Notably, we hypothesize that the effects of CAF are intricately linked to the nature of the task. It tends to amplify performance in simple cognitive tasks, but its impact is less obvious in more complex cognitive tasks. There is also a notable degree of variability in individual responses to CAF [59]. The effects of CAF can differ based on personal consumption patterns. For example, individuals who were not habituated to CAF intake had a more pronounced and lasting ergogenic impact than those with regular CAF consumption habits [62].

In discussing the undesirable effects of CAF supplementation, our findings revealed a positive correlation with dosage. Specifically, lower dosages were associated with fewer negative effects, whereas higher dosages were associated with a heightened incidence of adverse outcomes. This aligns with the findings of previous studies, reinforcing the notion that, regarding cognitive functioning and mood states, moderation is key. Smaller CAF dosages have been shown to elicit positive emotional responses, enhancing mental capabilities without precipitating stress [49]. Conversely, higher dosages have been linked to increased stress, irritability, and anxiety [49]. It has been observed that a smaller dose fosters positive emotions, unlike a larger dose that heightens feelings of tension, irritability, and nervousness [49]. Nevertheless, previous findings suggest that the intake of higher doses of CAF in the morning can lead to a significant incidence of undesirable effects, including nervousness, tachycardia, gastrointestinal issues, and notably, sleep disturbances, affecting approximately 35–40% of participants [46]. Thus, our study corroborates the idea that while CAF supplements can enhance cognitive performance, there is a delicate balance to avoid potential negative repercussions. Furthermore, recent studies indicate that both humans, especially females, and animals exhibit anxiety and similar behaviors after the administration of high doses of CAF [78,79]. The frequency of behaviors indicative of anxiety escalated when influenced by CAF, but these effects were somewhat diminished when fewer quantities of CAF were administered to animals [78]. Moreover, Paz-Graniel et al. [79] revealed that there is a direct correlation between increased CAF consumption and amplified instances of anxiety in women, a relationship not mirrored in men. Those with panic disorders, for instance, are more susceptible to anxiety and panic attacks triggered by CAF [80]. Although a comprehensive understanding of the mechanisms behind these effects has yet to be established, it is known that CAF exhibits high-affinity binding to adenosine A1 and A2AR receptors but binds with lower affinity to A2B and A3 receptors [81]. There are numerous proposed hypotheses attempting to explain the observed relationships between CAF consumption and generalized anxiety. The potential effects of CAF may be manifested through several mechanisms, including antagonizing adenosine receptors, inhibiting phosphodiesterase, promoting the release of intracellular stores/calcium, and opposing GABAA receptors [82]. Nevertheless, additional research is needed to validate and shed light on the potential cause-and-effect connection between the ingestion of CAF and the prevalence of anxiety.

Furthermore, the nocebo effect might contribute to the frequency of negative side effects observed when regular consumers of CAF decrease their intake [83]. In this context, while CAF has been shown to positively influence athletic performance, concerns have been raised regarding its usage among team sport athletes due to potential impacts on technical and tactical aspects, coupled with associated side effects such as nervousness, which could potentially lead to a decrease in accuracy performance [40]. The side effects observed in our study, particularly when higher doses of CAF were administered, along with the possibility of experiencing nocebo effects under placebo conditions, could potentially have adverse effects on athletic performance, particularly in scenarios involving successive competitions or matches during a short period. Notably, there is a scarcity of studies investigating the effects of CAF withdrawal on exercise performance, highlighting an urgent necessity for research in this domain to establish a viable restriction protocol [84]. Nonetheless, relatively little attention has been paid to the potential adverse effects of CAF consumption, particularly at the levels commonly used to enhance athletic performance [27]. Therefore, CAF administration should be carefully planned when it occurs during successive competitions or events.

Finally, various external factors, such as exercise, sex, and temperature, affect CAF metabolism, which mainly results in the production of paraxanthine, theobromine, and theophylline [85]. Previous research has shed light on the interaction between exercise, temperature, and caffeine pharmacokinetics in the body [86,87]. Studies have demonstrated that both acute and chronic exercise may alter CAF’s pharmacokinetic profile, notably increasing its maximum blood concentration while reducing its half-life and volume of distribution [86], without affecting clearance rates over eight hours, including the initial hour. Moreover, exercise influences the kinetics of theophylline, a related methylxanthine, with its half-life extending, and a notable decrease in volume of distribution, especially during moderate and light exercise under hot conditions [87]. McLean and Graham [85] suggested that exercise modifies methylxanthine metabolism but did not report any metabolic changes when controlling for variables such as menstrual cycle, gender, exercise, and thermal stress, as well as reproductive status and environmental or dietary factors. 

Concerning gender differences in CAF’s effects, a previous study indicates that there might be a decrease in the performance-enhancing benefits of CAF due to its interaction with female hormones [88]. However, other findings reveal consistent absorption and immediate effects of CAF across different menstrual phases, with female athletes benefiting from CAF consumption irrespective of their menstrual cycle phase [89,90]. Additionally, the metabolism of CAF has been shown to interact with oral contraceptive use, potentially prolonging its effects [91]. Our study excluded female athletes using contraception to maintain result consistency, a factor rarely considered in similar research [84,92]. To the best of our knowledge, no previous studies have directly compared the effects of CAF intake on cognitive performance across different temperature contexts, or among athletes of both sexes, highlighting the necessity for future research. This gap underscores the challenge of generalizing findings from male to female athletes or across various athletic contexts.

This line of investigation is essential for future studies, as certain factors could modify the pharmacokinetics of CAF, possibly affecting the dosage required to improve cognitive performance and influencing any side effects associated with the timing of CAF intake. Previous studies have explored the relationship between timing and CAF supplementation, revealing that morning CAF intake effectively counteracts the decline in cognitive and physical performance observed in the morning [93,94]. Nevertheless, afternoon CAF consumption was associated with significant adverse effects like anxiety and insomnia and did not improve the athletes’ physical or cognitive performance [93,94]. A recent study by Bougrine et al. [21] suggested that female athletes may benefit from caffeine intake in the morning but not in the afternoon. The study observed a trend where the negative side effects of moderate CAF intake (6 mg·kg^−1^) were less noticeable in the morning but became more pronounced in the afternoon without corresponding enhancements in cognitive and physical parameters. Moreover, with the same dose of CAF, Mora-Rodríguez et al. [95] and Dunican et al. [22] reported that evening CAF intake has minimal impact on neuromuscular performance. Thus, attention should be paid to the use of this substance when two games are played on consecutive days or when training routines are carried out in the afternoon. This consideration is key to maximizing the benefits and minimizing the potential negative effects of this stimulant. The results of this study have significant practical implications for athletes in sports where quick decision-making and sharp attention are critical to optimizing cognitive performance through dietary supplementation.

### Strength and Limitations

A significant strength of the current research is the investigation of various dosage effects of CAF on a range of cognitive tasks within the same demographic, particularly including female athletes, who are often under-represented in depth within such studies. Despite this, our study faced several limitations. It is important to note that the conclusions drawn from our population of healthy, athletic women may not extend to non-athlete populations due to varying levels of fitness or to male subjects because of inherent physiological and physiological differences between sexes. Additionally, while we analyzed cognitive performance only at rest, evaluating cognitive performance during physical exercise remains a pertinent area for further investigation to aid athletes performing concurrent physical and cognitive tasks. Our selection criteria limited participants to low CAF consumers, suggesting that habitual CAF intake may modulate the ergogenic effects observed. Further studies among female low consumers are needed, particularly those adhering to recent CAF user classifications [32]. We also acknowledge that, despite aligning with the sample size calculation recommendation to achieve a strong statistical power of 0.95, the enrolment of only 14 female athletes in our study represents a relatively small sample size. This limitation significantly restricts the ability to generalize our findings. Finally, the absence of blood sample analysis precluded us from confirming peak serum CAF levels or whether differential doses produced variable serum concentrations. Further research is warranted to optimize pre-exercise CAF dosing to enhance cognitive function, considering factors such as athlete training levels, habitual CAF use, and the types of cognitive tasks employed.

## 5. Conclusions

The current study established that the ingestion of CAF at doses of 3 and 6 mg·kg^−1^ led to improvements in simple and choice reaction time and attentional task performance, with a lower dosage yielding more significant enhancements. Interestingly, mental rotation performance did not benefit from CAF supplementation, regardless of the dose. Notably, a higher dosage of CAF (9 mg·kg^−1^) was linked to an increase in adverse side effects without any beneficial effects on cognitive tasks. These findings suggest that low-dose CAF consumption, specifically at 3 mg·kg^−1^, is safer and more effective at improving simple cognitive functions than moderate or high doses, indicating the potential of low-dose CAF as a targeted supplement for enhancing simple cognitive tasks. 

## Figures and Tables

**Table 1:** Side effects reported by the athletes immediately after the conclusion of each cognitive test battery (Q + 0 h) and 24 h later (Q + 24 h). The data are presented as percentages of prevalence (%).

	PLAC		3 mg of CAF		6 mg of CAF		9 mg of CAF	
	Q + 0 h	Q + 24 h	Q + 0 h	Q + 24 h	Q + 0 h	Q + 24 h	Q + 0 h	Q + 24 h
Muscle soreness	0	7.14	0	7.14	7.14	7.14	7.14	14.28
Increased urine output	0	7.14	0	7.14	0	14.28	0	35.71
Tachycardia	7.14	0	14.28	14.28	21.42	14.28	35.71	35.71
Anxiety or nervousness	0	0	0	0	0	7.14	7.14	21.42
Headache	7.14	0	7.14	7.14	7.14	14.28	21.42	28.57
Gastrointestinal problems	0	7.14	0	0	12.5	0	28.57	35.71
Insomnia	-	0	-	0	-	14.28	-	28.57
Increased vigor/activeness	7.14	0	7.14	0	14.28	7.14	14.28	7.14
Perception of performance improvement	7.14	-	14.28	-	14.28	-	14.28	-

mg: milligram and CAF: Caffeine.

## Data Availability

All participants provided signed consent for the publication of the study findings. The data from this study are available upon reasonable request from the first author.
